# Effect of cold spells and their modifiers on cardiovascular disease events: Evidence from two prospective studies

**DOI:** 10.1016/j.ijcard.2016.05.012

**Published:** 2016-09-01

**Authors:** Claudio Sartini, Sarah J.E. Barry, S. Goya Wannamethee, Peter H. Whincup, Lucy Lennon, Ian Ford, Richard W. Morris

**Affiliations:** a Department of Primary Care & Population Health, University College London, Rowland Hill Street, NW3 2PF London. UK; b Institute of Health and Wellbeing, College of Medical, Veterinary and Life Sciences, Robertson Centre for Biostatistics, Boyd Orr Building, Level 11 University of Glasgow, Glasgow G12 8QQ, UK; c Population Health Research Institute, St George's University of London, Cranmer Terrace, London SW17 0RE, UK; d School of Social & Community Medicine, University of Bristol, Canynge Hall, 39 Whatley Rd, Bristol BS8 2PS, UK

**Keywords:** Cold spell, Outdoor temperature, Winter deaths, Cardiovascular disease, Prospective study, Older people

## Abstract

**Objective:**

To investigate effects of cold weather spells on incidence of cardiovascular disease (CVD), and potential effect modification of socio-demographic, clinical, behavioural and environmental exposures.

**Methods:**

Data from two prospective studies were analysed: the British Regional Heart Study (BRHS), a population-based study of British men aged 60–79 years, followed for CVD incidence from 1998–2000 to 2012; and the PROSPER study of men and women aged 70–82 recruited to a trial of pravastatin vs placebo from 1997 to 9 (followed until 2009). Cold spells were defined as at least three consecutive days when daily mean temperature fell below the monthly 10th percentile specific to the closest local weather station. A time-stratified case-crossover approach was used to estimate associations between cold spells and CVD events.

**Results:**

921 of 4252 men from BRHS and 760 of 2519 participants from PROSPER suffered a first CVD event during follow-up. More CVD events were registered in winter in both studies. The risk ratio (RR) associated with cold spells was statistically significant in BRHS (RR = 1.86, 95% CI 1.30–2.65, p < 0.001), and independent of temperature level: results were similar whether events were fatal or non-fatal. Increased risk was particularly marked in BRHS for ever-smokers (RR of 2.44 vs 0.99 for never-smokers), in moderate/heavy drinkers (RR 2.59 vs 1.41), and during winter months (RR 3.28 vs 1.25). No increased risk was found in PROSPER.

**Conclusions:**

Although CVD risks were higher in winter in both BRHS and PROSPER prospective studies, cold spells increased risk of CVD events, independently of cold temperature, in the BRHS only.

## Highlights

• CVD risks were higher in winter in both BRHS and PROSPER studies of older adults.
• In BRHS, we found an added impact of cold spells on CVD, independent of temperature.
• There was an increased susceptibility to cold spells among smokers and drinkers.
• Findings support UK guidelines on identification of vulnerable older people.

## 1 Background

Cardiovascular disease (CVD) is the most common cause of death globally, remaining a considerable burden both in terms of health and costs [1]. As in many countries, CVD mortality in the UK exhibits a marked seasonal variation; more people die during the winter months (December–March) than in other periods of the year and the majority of deaths occur among those aged 75 and over [2], [3]. This seasonal variation in death rates has been mainly attributed to cold weather and fall in temperature, which can alter vulnerability to specific diseases, in particular myocardial infarction, stroke and respiratory infection (especially influenza) [4], [5], [6], [7]. However, uncertainty still exists about the range in temperature which produces an increased risk of CVD and other health outcomes, [8], [9] since effects of both extremely cold days [10], [11] and moderately cold days [8] on mortality have been demonstrated. To date, there is neither an established definition of a cold day nor a precise definition of the period for which a cold spell (e.g. two or more consecutive cold days) should last for detrimental health effects [9]. Less frequently, cold spells in the UK can also occur during the non-winter months (May–November) [12], with lowest minimum and maximum temperatures in England of − 2 °C and 9 °C in August [13].

A much debated question is which people are more susceptible to cold temperature or cold spells, and the relative importance of individual characteristics such as age, previous chronic conditions, low income and cold homes [7], [14], [15], [16], [17]. The elderly have been long considered more susceptible to cold weather [5], but the evidence is not consistent [17], For example, the odds of death in the elderly may be significant only if associated with cold spells, but not a linear decrease in temperature [15]. In other studies the statistical power to examine evidence for effect modification was low and evidence for differences in effect of cold temperature on cardiovascular mortality according to obesity, smoking habit, alcohol intake, and hypertension was not found [16].

Therefore, the aims of this study are threefold: (i) to investigate the effect of cold spells on cardiovascular events during 1997–2012 (subdivided into fatal and non-fatal, and coronary and stroke) using data from two large prospective studies of older adults; (ii) to explore whether the effect of cold spells is modified by established cardiovascular risk factors (e.g. age and smoking) and previously unexplored individual characteristics (e.g. physical activity score, central heating and double glazing in the house); (iii) to explore whether the effect of cold spell is independent from average temperature over periods up to 6 days previously.

We carried out a primary analysis on men from an established UK population-based study, the British Regional Heart Study (BRHS) [18], and secondarily on participants of the PROspective Study of Pravastatin in the Elderly at Risk (PROSPER) [19], [20] recruited from Glasgow (UK), Cork (Republic of Ireland), Leiden (The Netherlands) and the surrounding areas.

## 2 Methods

### 2.1 Methods and participants

Participants from BRHS and PROSPER provided informed written consent, which was performed in accordance with the principles of the Declaration of Helsinki. The designs of both BRHS [21] and PROSPER [19], [20], which are both prospective studies of several thousand participants with cardiovascular disease as their key endpoints, have been previously described in detail and included in this work as supplementary material (Supplementary File 1 – BRHS and PROSPER methods and participants).

### 2.2 Case ascertainment and follow-up

The BRHS cohort was followed-up from Jan. 1998–March 2000 until the end of 2012, while the PROSPER participants were followed-up from December 1997–May 1999 until the end of June 2009. The events considered during the corresponding study periods for the two studies were fatal or nonfatal stroke and CHD death or non-fatal myocardial infarction (MI). The definitions of non-fatal/fatal stroke and CHD death/non-fatal MI are reported in supplementary material (Supplementary File 1 — Definition of fatal and non-fatal CVD events).

### 2.3 Climatic data and definition of cold spell

Mean temperature of the day for the study towns was provided by the national meteorological offices (Supplementary File 1 — Climatic data). The definition of cold spell used in this study was derived from daily mean temperatures and related to spells which were, for at least 3 or 4 consecutive days, below the 10th percentile for that geographical location for the specific month of the year (for details see Supplementary File 1 — Definition of cold spell).

### 2.4 Statistical methods

Firstly, baseline characteristics of BRHS and PROSPER participants were compared between those who did or did not experience the CVD events (non-fatal or fatal) during follow-up.

Then, monthly descriptive statistics of number of events were calculated. Average mean temperatures, and number of cold spells of at least 3 and 4 consecutive days were calculated by month, for both BRHS and PROSPER separately, and during event days and control days (defined below).

Only participants who suffered an event were included in subsequent analysis, and short-term associations between cold spell and CVD events were assessed using a time-stratified case-crossover approach, widely used in environmental epidemiology [22]. A case-crossover study can be seen as a self-matched case–control study: for each individual, exposure data are collected for the “case” day (that is, the day of the cardiovascular event) and a set of “control” days that were not associated with the event of interest. The “control” days were selected by using the same days of the week of the same month and year [23]. For each “case” and “control” day we determined whether the specific day and days preceding were cold days.

We then compared cases with their set of controls using conditional logistic regression. The outcome was a binary variable (case or control) as was the exposure variable (day of event being part of a cold spell or not). Therefore, the odds ratios from the conditional-logistic-regression model can be interpreted as risk ratios (RRs). By design, the analyses are adjusted for long-term changes in environmental exposures, for day of the week, and for all participant characteristics that are expected to remain stable over a 1-month period (e.g., smoking status).

We reported results for 7 different outcomes: (1) all causes of death; (2) fatal events (fatal stroke or CHD death); (3) CHD death; (4) fatal stroke; (5) earliest of fatal/non-fatal stroke or CHD death/non-fatal MI; (6) MI events (earliest nonfatal MI or CHD death); (7) stroke events (earliest non-fatal or fatal) stroke. For each outcome type, only the first event of the relevant type was included. Results are presented separately for the two studies but we also carried out a fixed effects meta-analysis to pool results.

We made use of the wide range of individual risk factors in BRHS to examine interaction effect between cold spells and these risk factors.

## 3 Results

### 3.1 Participants' characteristics

In the BRHS, 4252 men out of 5875 men (72.4%) were alive at 01/01/1998 and participated at the 20 year follow-up examination and survey. 3977 men (67.7%) did not change town of residence during the study period 1998–2012. Participants were followed for a median of 12.7 years (inter-quartile range 8.7, 13.5). The BRHS participants' characteristics are shown in Table 1a, according to whether or not they later experienced CVD events (921 participants: 23.2%, and 521 (13.1%) fatal events (fatal stroke or CHD death). The baseline characteristics of the study participants for PROSPER are reported in Table 1b. In the PROSPER study non-fatal events were available for the Glasgow Centre only: 760 out of 2520 participants (30.2%) had development of CVD (earliest of fatal or non-fatal stroke or non-fatal MI or CHD death). Considering all PROSPER cohorts (Glasgow plus Cork and Leiden), the number of CVD deaths (fatal stroke or CHD death) registered during the follow up period (median = 10.3 years (IQR 6.9 to 10.7) was 810 out of 5804 (14%).

PROSPER participants in comparison with BRHS participants (see Table 1a vs Table 1b) were about 5 years older, with a higher CVD prevalence, but also less likely to have diabetes, to be smokers, or to drink alcohol. PROSPER participants were also more likely to live alone and use aspirin, beta-blockers, ACE-inhibitors, diuretics, calcium channel blockers and nitrates. Owing to the nature of the PROSPER study, 50% were initially assigned to statins, while less than 10% of BRHS participants took statins at baseline.

### 3.2 Monthly distribution of events, temperatures and cold spells

During the study period, mortality from all causes and from CVD (fatal stroke or CHD death) was highest during the winter months (December–March) in both BRHS (Table 2a) and PROSPER (Table 2b) cohorts, see also eFigure 1 (Supplementary material). The excess winter mortality (EWM) from all causes of death was 18% in both studies, but the EWM from CVD was higher (36% in BRHS and 23% in PROSPER).

Mean temperatures on event or control days (where ‘events’ were first events of any type; ie. outcome 5 as defined in the Methods section) were lowest from December to March in both BRHS (Table 2a) and PROSPER (Table 2b).

Cold spells of both ≥ 3 days and ≥ 4 days were more common during winter (December–March). More cold spells occurred during follow-up of BRHS participants (1998–2012) than of PROSPER participants (1997–2009). During the BRHS follow-up cold spells were more common during events days than control days (p < 0.001 for BRHS), while there was no evidence of a difference in PROSPER (p = 0.933 for PROSPER).

### 3.3 Associations (main effect) between cold spells and cardiovascular events

Using the time-stratified case-crossover approach, associations were noted between cold spells of ≥ 3 days and ≥ 4 days and some end-points (CVD mortality and development of CVD events) in the BRHS study (Table 3). Associations were found between cold spells of ≥ 3 days and ≥ 4 days and the following end-points: (i) fatal events (fatal stroke or CHD death); (ii) CHD death; (iii) earliest of fatal/non-fatal stroke or CHD death/non-fatal MI; (iv) MI events (earliest nonfatal MI or CHD death). Risk ratios were stronger for cold spells of ≥ 4 days than of ≥ 3 days and independent of mean temperature over the previous 6 days (lag 0–6). Estimates were similar and still significant when cold spells of ≥ 4 days was adjusted for mean temperature on the day itself (lag 0), over the previous day (lag 0–1) and the previous three days (lag 0–3). Therefore, Table 3 reports the estimates adjusted for temperature over the previous 6 days only.

No associations were found between cold spells and any of the various CVD end-points during the PROSPER follow-up period. However, there was weak evidence that cold spells of ≥ 3 days affected all-cause mortality [RR = 1.28 (0.99, 1.65)].

Pooling the effects on non-fatal or fatal events across studies yielded an odds ratio of 1.50 (95% CI 1.11 to 2.02, p = 0.008): however there was evidence of heterogeneity between studies (I^2^ = 78%, p = 0.03).

### 3.4 Associations (main effect) between mean temperature and cardiovascular events

Associations were noted between mean temperature and development of CVD events in the BRHS study only (Table 4). Associations were found between a decrease in mean temperature, at both lag 0–3 and lag 0–6, and the following end-points: (i) earliest of fatal/non-fatal stroke or CHD death/non-fatal MI; (ii) MI events (earliest non-fatal MI or CHD death), and (iii) stroke events (earliest non-fatal or fatal stroke). Moreover, the effect of temperature at lag 0–3 was independent of cold spells (≥ 3 days) for (i) earliest of fatal/non-fatal stroke or CHD death/non-fatal MI, and (iii) stroke events (earliest non-fatal or fatal stroke).

No associations were found between temperature and the different end-points during the PROSPER follow-up period.

### 3.5 BRHS follow-up: interactions between cold spells effect and individual risk factors

Table 5 shows the interactions between cold spells of different durations and individual risk factors on earliest of either non-fatal/fatal MI/Stroke for BRHS only. There were suggestions of increased susceptibility to cold spells (of both ≥ 3 and ≥ 4 days of duration) in relation to smoking status: BRHS men experiencing a cold spell who were current/former smokers showed a higher risk of a CVD event than never smokers (2.79 vs 0.58, interaction test: p = 0.019 for cold spell ≥ 4 days of duration; 2.44 vs 0.97, interaction test: p = 0.034 for cold spell ≥ 3 days of duration). Moreover, there were suggestions of increased susceptibility to cold spells for the following groups: men who consumed alcohol more than occasionally vs others (OR 3.29 vs 1.34, interaction test: p = 0.039 for cold spell of ≥ 4 days), car owners (OR 2.44 vs 0.94, interaction test: p = 0.035 for cold spell of ≥ 3 days), and men who developed an event in winter, between December and March (RR = 3.28 vs 1.25, interaction test: p = 0.004 for cold spell of ≥ 3 days).

Given the differences in effects of cold spells between BRHS and PROSPER, and the vast difference in initial statin usage between the two studies, we investigated whether statin users seemed to be protected from cold spells. There was no evidence of interaction between cold spells and statin use on CVD events in either study. However there was weak evidence of interaction for all-cause mortality in PROSPER with the relative risk for 3-day cold spells being 1.28 for those assigned to placebo and 0.69 for those assigned to statins (p-for-interaction = 0.043).

## 4 Discussion

Using data from the British Regional Heart Study (BRHS), we have demonstrated the effect of cold spells of weather over at least 3 or 4 days on incidence of cardiovascular disease. A 4 day spell increased risk more than two-fold. This finding was independent of the actual temperature level over periods up to two weeks prior to an event occurring. Effects appeared fairly similar among all subdivisions of the endpoint, including stroke and coronary events, both fatal and non-fatal, but were particularly strong for coronary heart disease events. Furthermore we found that the strength of the effect was greater among smokers, possibly among those with moderate or greater alcohol consumption, and during winter months. The effect appeared to be greater among those who owned a car.

The analysis of the BRHS included men whose age at baseline ranged from 60 to 79 years and were followed for an average exceeding 12 years. The men lived across a full geographical range of Great Britain (England, Wales and Scotland), representing the well-documented national variation in cardiovascular mortality rates [18]. Analysis was based on CVD events in 921 participants, and this gave sufficient statistical power to demonstrate increased risk following three or four day cold spells. Power to demonstrate effect modification was however limited and while some factors were shown to increase the magnitude of the effect of cold weather spells, these barely reached statistical significance at the 5% level. Of note, despite repeated consistent findings that older people have sharper excess winter mortality than younger people [5], the expected differences in relative risks between younger and older men in BRHS did not emerge as statistically significant.

Those factors where suggestion of effect modification was closest to statistical significance included smoking and alcohol consumption. Smoking and alcohol intake may have enhanced vaso-constriction and led to blood pressure changes, which have been suggested as partly responsible for excess winter mortality from cardiovascular disease [24], [25]. Participants who owned a car appeared particularly susceptible to cold weather in comparison with participants who were not car owners. However in general car ownership was associated with lower CVD incidence in BRHS: men who owned a car were more likely to be of non-manual social class, younger, employed rather than retired, and more physically active; although more likely to use aspirin. Individual exposure to traffic air pollution data was not available in this study and it is unclear whether or not this may have an impact on those who developed an event. Finally, although the effect of cold spells appeared to operate independently from that of cold temperature, it was clear that cold spells had more effect in winter months.

We carried out similar analysis on data from the PROSPER study which included men and women aged over 70 at baseline. Similarly to the BRHS and accordingly with previous findings and national mortality statistics [4], a higher number of fatal CVD events and deaths from all-causes were registered during winter months in PROSPER. However, in PROSPER neither effect of cold spells nor of average temperature was observed on the outcomes. By adding PROSPER data to that of BRHS it was hoped to confirm and extend the BRHS findings using a large study of older adults residing in three areas inside and outside the UK but with similar latitude and climate (Glasgow, Cork, and Leiden). The PROSPER analysis would have allowed estimation of the effect of cold spells with greater precision, and test whether effects held for women as well as men. However no associations between cold spells and CVD events were seen in PROSPER either in men or women, and these results differed significantly from BRHS. By study design, the PROSPER study excluded people who were abusers of alcohol [19] and the resulting level of alcohol use was lower among PROSPER participants than in BRHS. The smoking rate was also lower and it is possible that the PROSPER participants were thus less susceptible to CVD events following cold spells. Several cardiovascular medications were used more frequently by PROSPER participants but the greatest difference was in statin use, as 50% of PROSPER participants were assigned to pravastatin as part of the trial, whereas less than 10% of BRHS participants reported statin use at baseline. However in neither study was a significant interaction in the effect of cold spells on CVD events according to statin usage.

### 4.1 Strengths

To our knowledge, this is the first analysis that has employed the concept of cold spells as opposed to temperature levels. Given the repeated finding that excess winter mortality is seen even in countries with relatively warm climate [4], [6], [8], we hypothesised that relative cold was more important than absolute cold. It was therefore possible that temperature that was cold for the time of year had more impact. We have demonstrated in the BRHS that although temperature level up to 6 days carried some impact on CVD risk, there was an added impact of cold spells over 3 or 4 days. Moreover, a main strength was the analysis of the effect of cold spells by established cardiovascular risk factors (e.g. age and smoking) and previously unexplored individual characteristics (e.g. physical activity score, central heating and double glazing in the house).

### 4.2 Limitations

Not all fatal events in BRHS or PROSPER represented sudden deaths and for some individuals, a non-fatal event may still have occurred up to 28 days previously [19], [26], [27]. The exact date of such events was not known and thus our analysis refers to weather at the time of the death rather than the weather at the event which preceded it. This might have led to a diluted estimate of the true association between cold spells and CVD incidence, especially when analysing fatal events alone. However a Swedish study has suggested that a minority of fatal CHD events occur inside hospital and this proportion has decreased over time [28].

Compared to the BRHS, the exposure assessment to outdoor temperature in PROSPER was less accurate for two reasons: (i) PROSPER participants were recruited from wide areas around Glasgow, Cork and Leiden, and matched with the only weather station available in town (while in the BRHS the participants living in 24 UK towns and their surroundings were matched with the closest of 35 weather stations via postcode); (ii) as postcode of residence was not available in PROSPER, it is not known whether or not the participants changed town of residence or sub-region to during the study period (differently, only BRHS participants who did not change postcode of residence were included). Therefore, a misclassification of exposure to outdoor temperature was possible and the estimates may be less accurate in PROSPER.

### 4.3 Implications

Our study confirmed an excess of winter mortality from CVD and also susceptibility to cold spells in the BRHS population. The role of cold spells and cold weather continue to be debated, and this study added new evidence that vulnerable subgroups of older men may be particularly exposed to the effects of cold weather, and prolonged spells of unusual degrees of cold for the time of year might be particularly hazardous. Recent guidance from the UK National Institute for Health Care and Excellence [29] made a key recommendation concerning the establishment of a single point-of-contact referral service for vulnerable older people living in cold homes. Primary care team practitioners (including GPs, community matrons and district nurses) as well as social care professionals have been asked to identify the heating needs of vulnerable people and refer them where appropriate. However the means by which the recommendation can practically be achieved now needs to be evaluated for its impact on the UK's persisting excess winter mortality.

## 5 Conclusions

A higher number of CVD events occurred during winter months in both BRHS and PROSPER prospective studies. However, cold spells increased risk of CVD events, and independently of cold temperature, in the BRHS only, a population-based study representative of older men in Great Britain. Some health behaviours may have made BRHS men more susceptible. Strategies to avoid excess winter mortality due to CVD should account for the impact of generally low temperature coupled with particularly cold spells. This study also highlighted the importance of accurate assessment of exposure to outdoor temperature, for more accurate estimates of a cold spell effect.

## Authors' contributions

CS processed the BRHS data, performed statistical analyses, drafted and revised the manuscript, and incorporated revisions of co-authors, and approved the final version.

SB processed the PROSPER data, performed statistical analyses, revised the manuscript, and approved the final version.

GSW, PHW, and IF contributed to the design of the study, to the acquisition of data, revised the manuscript, and approved the final version.

LL enrolled BRHS participants, collected data, and approved the final version.

RWM raised grant funding, contributed to the design of the study, to the acquisition of data, to the supervision of statistical analyses, drafted and revised the manuscript, and approved the final version.

## Conflict of interest statement

The authors report no relationships that could be construed as a conflict of interest.

## Figures and Tables

**Table 1a:** BRHS participant's characteristics (January 1998–March 2000) subdivided by those who did or did not experience CVD events (non-fatal or fatal) during follow-up (January 1998–December 2012).

	Have had fatal/non-fatal CVD event (non-fatal/fatal stroke or non-fatal MI/CHD death)		CVD death (fatal stroke or CHD death)	
	Yes (n = 921)	No (n = 3056)	Yes (n = 521)	No (n = 3456)
Demographic and background characteristics				
Age (years), mean (SD)	70.4(5.5)	68.2(5.4)	71.8(5.2)	68.2(5.4)
Social class (manual), n(%)	488(53.2)	1565(51.2)	289(55.6)	1764(51.1)
Physical health				
Prevalence of non-fatal stroke or MI at baseline, n(%)	158(17.2)	193(6.3)	115(22.1)	236(6.8)
BMI, mean (SD)	27.1(3.8)	26.9(3.7)	27.0(3.8)	27.0(3.7)
Diabetes, n(%)	137(14.9)	309(10.1)	82(15.7)	364(10.5)
Lung function (FEV_1_/FVC < 70%), n(%)	242(26.7)	792(26.2)	151(29.5)	883(25.8)
Behaviour				
Physical activity score at baseline				
Inactive, n(%)	145(16.5)	301(10.2)	97(19.7)	349(10.4)
Occasional/Light, n(%)	380(43.3)	1228(41.5)	208(42.3)	1400(41.9)
From moderately to vigorously active, n(%)	352(40.1)	1427(48.3)	187(38.0)	1592(47.7)
Smoking				
Never, n(%)	233(25.4)	908(29.7)	113(21.8)	1028(29.8)
Former, n(%)	548(59.8)	1754(57.5)	316(60.9)	1986(57.5)
Current, n(%)	136(14.8)	391(12.8)	90(17.3)	437(12.7)
Alcohol consumption				
None, n(%)	114(12.6)	287(9.5)	66(13.0)	335(9.8)
Occasional (< 1 drink/week), n(%)	276(30.6)	775(25.7)	158(31.1)	893(26.2)
Light (1–15/week), n(%)	344(38.1)	1363(45.3)	196(38.6)	1511(44.3)
Moderate/regular (16–42/weeks), n(%)	133(14.7)	480(15.9)	68(13.4)	545(16)
Heavy (> 42/week), n(%)	29(3.2)	92(3.1)	16(3.1)	105(3.1)
Unclassified, n(%)	7(0.8)	15(0.5)	4(0.8)	18(0.5)
House/accommodation				
Owner occupier, are you:				
An owner occupier, n(%)	745(83.7)	2621(87.9)	408(81)	2958(87.8)
Does your home have				
Central heating (yes), n(%)	807(92.0)	2742(93.5)	447(90.9)	3102(93.5)
Double glazing (yes/in part), n(%)	517(85.9)	1648(85.4)	286(83.6)	1879(85.8)
Personal circumstances				
Marital status				
Married (yes), n(%)	721(81.8)	2525(84.9)	392(78.7)	2854(85)
Working status				
Retired, n(%)	778(86.6)	2351(78.6)	467(92.5)	2662(78.7)
Living conditions				
Living alone, n(%)	128(14.3)	325(11)	88(17.3)	365(10.9)
Car ownership (Yes/No)				
Yes, n(%)	705(78.4)	2549(84.5)	370(72.7)	2884(84.7)
Use of medication (yes)				
Anti-coagulants, Warfarin, n(%)	44(4.8)	73(2.4)	32(6.1)	85(2.5)
Aspirin, n(%)	358(39.1)	719(23.6)	223(43.2)	854(24.8)
Beta-adrenoceptor blocking drugs, n(%)	150(16.3)	389(12.7)	95(18.2)	444(12.8)
Statin, n(%)	101(11.0)	202(6.6)	63(12.1)	240(6.9)
ACE-Inhibitors, n(%)	7(0.8)	9(0.3)	5(1.0)	11(0.3)
A-II receptor antagonists, n(%)	5(0.5)	16(0.5)	3(0.6)	18(0.5)
Diuretics, n(%)	181(20.5)	356(10.9)	128(25.9)	409(11.2)
Calcium channel blockers, n(%)	178(20.1)	402(12.4)	118(23.8)	462(12.7)
Nitrates, n(%)	138(15.6)	236(7.3)	92(18.6)	282(7.7)
Other antihypertensive, n(%)	2(0.2)	7(0.2)	2(0.4)	7(0.2)
Blood glucose lowering: insulin, n(%)	17(1.9)	26(0.8)	10(2.0)	33(0.9)
Blood glucose lowering: oral hypoglycaemics, n(%)	54(6.1)	102(3.1)	35(7.7)	121(3.3)
Established CVD risk factors				
Systolic blood pressure, mm Hg, mean (SD)	153.3(26.1)	147.8(23.6)	154.3(27.5)	148.3(23.7)
Diastolic blood pressure, mm Hg, mean (SD)	85.7(12.2)	85.0(10.8)	85.3(12.9)	85.1(10.9)
Total cholesterol, mmol/L, mean (SD)	6.0(1.1)	6.0(1.1)	6.0(1.0)	6.0(1.1)
LDL-cholesterol, mmol/L, mean (SD)	3.9(1.0)	3.9(1.0)	3.9(1.0)	3.9(1.0)
HDL-cholesterol, mmol/L, mean (SD)	1.3(0.3)	1.3(0.3)	1.3(0.3)	1.3(0.3)
Triglycerides, mmol/L, mean (SD)	1.9(1.1)	1.8(1.1)	1.9(1.1)	1.8(1.1)

**Table 1b:** PROSPER participant's characteristics (December 1997 – May 1999) subdivided by those who did or did not experience CVD events (non-fatal or fatal) during follow-up (December 1997–June 2012).

	Have had fatal/non-fatal CVD event (non-fatal/fatal stroke or non-fatal MI/CHD death), Glasgow participants		CVD death (fatal stroke or CHD death), all PROSPER participants	
	Yes (n = 760)	No (n = 1760)	Yes (n = 810)	No (n = 4994)
Demographic and background characteristics				
Age (years), mean (SD)	75.9 (3.4)	75.0 (3.3)	76.3 (3.4)	75.2 (3.3)
Social class (manual), n(%)	n.a.	n.a.	n.a.	n.a.
Physical health				
Prevalence of non-fatal stroke or MI at baseline, n(%)	185 (24.3)	278 (15.8)	223 (27.5)	756 (15.1)
BMI, mean (SD)	26.8 (4.4)	26.7 (4.2)	26.6 (4.2)	26.9 (4.2)
Diabetes, n(%)	85 (11.2)	128(7.3)	99 (12.2)	524 (10.5)
Lung function (FEV_1_/FVC < 70%), n(%)	n.a.	n.a.	n.a.	n.a.
Behaviour				
Physical activity score at baseline				
Inactive, n(%)	n.a.	n.a.	n.a.	n.a.
Occasional/Light, n(%)	n.a.	n.a.	n.a.	n.a.
From moderately to vigorously active, n(%)	n.a.	n.a.	n.a.	n.a.
Smoking				
Never, n(%)	205 (27.0)	586 (33.3)	238 (29.4)	1731 (34.7)
Former, n(%)	340 (44.7)	681 (38.7)	357 (44.1)	1920 (38.4)
Current, n(%)	215 (28.3)	493 (28.0)	215 (26.5)	1343 (26.9)
Alcohol consumption				
None, n(%)	352 (46.3)	841 (47.8)	378 (46.7)	2198 (44.0)
Occasional (< 1 drink/week), n(%)	168 (22.1)	406 (23.1)	168 (20.7)	1175 (23.5)
Light (1–15/week), n(%)	239 (31.4)	511 (29.0)	260 (32.1)	1612 (32.3)
Moderate/regular (16–42/weeks), n(%)	1 (0.1)	2 (0.1)	4 (0.5)	9 (0.2)
Heavy (> 42/week), n(%)	0 (0)	0 (0)	0 (0)	0 (0)
Unclassified, n(%)	0 (0)	0 (0)	0 (0)	0 (0)
House/accommodation				
Owner occupier, are you:				
An owner occupier, n(%)	n.a.	n.a.	n.a.	n.a.
Does your home have				
Central heating (yes), n(%)	n.a.	n.a.	n.a.	n.a.
Double Glazing (yes/in part), n(%)	n.a.	n.a.	n.a.	n.a.
Personal circumstances				
Marital status				
Married (yes), n(%)	n.a.	n.a.	n.a.	n.a.
Working status				
Retired, n(%)	n.a.	n.a.	n.a.	n.a.
Living conditions				
Living alone, n(%)	350 (46.1)	747 (42.4)	329 (40.6)	1988 (39.8)
Car ownership (Yes/No)				
Yes, n(%)	n.a.	n.a.	n.a.	n.a.
Use of medication (yes)				
Anti-coagulants, Warfarin, n(%)	7 (0.9)	7 (0.4)	6 (0.7)	31 (0.6)
Aspirin, n(%)	347 (45.6)	613 (34.8)	390 (48.1)	1714 (34.3)
Beta-adrenoceptor blocking drugs, n(%)	201 (26.4)	412(23.4)	229 (28.3)	1273 (25.5)
Statin, n(%)	369 (48.6)	891 (50.6)	392 (48.4)	2499 (50.0)
ACE-inhibitors, n(%)	82 (10.8)	184 (10.4)	139 (17.2)	812 (16.3)
A-II receptor antagonists, n(%)	13 (1.7)	13 (0.7)	21 (2.6)	95 (1.9)
Diuretics, n(%)	316 (41.6)	729 (41.4)	350 (43.2)	2008 (40.2)
Calcium channel blockers, n(%)	247 (32.5)	537 (30.5)	246 (30.4)	1212 (24.3)
Nitrates, n(%)	247 (32.5)	364 (20.7)	248 (30.6)	843 (16.9)
Other antihypertensive, n(%)	29 (3.8)	53 (3.0)	41 (5.1)	196 (3.9)
Blood glucose lowering: insulin, n(%)	5 (0.7)	7 (0.4)	7 (0.9)	44 (0.9)
Blood glucose lowering: oral hypoglycaemics, n(%)	47 (6.2)	62 (3.5)	56 (6.9)	303 (6.1)
Established CVD risk factors				
Systolic blood pressure, mm Hg, mean (SD)	155.5 (22.4)	152.7 (20.7)	155.1 (23.2)	154.6 (21.6)
Diastolic blood pressure, mm Hg, mean (SD)	82.7 (11.5)	82.6 (10.6)	82.3 (12.0)	84.0 (11.3)
Total cholesterol, mmol/L, mean (SD)	5.63 (0.93)	5.72 (0.95)	5.67 (0.88)	5.68 (0.91)
LDL-cholesterol, mmol/L, mean (SD)	3.78 (0.80)	3.83 (0.83)	3.80 (0.80)	3.79 (0.80)
HDL-cholesterol, mmol/L, mean (SD)	1.25 (0.34)	1.31 (0.36)	1.25 (0.35)	1.28 (0.35)
Triglycerides, mmol/L, mean (SD)	1.58 (0.70)	1.56 (0.70)	1.55 (0.68)	1.54 (0.71)

**Table 2a:** BRHS events, temperature, and cold spells by month.

	Events							Temperature (°C)		Cold spell 3 + days		Cold spell 4 + days	
BRHS follow up period: from Jan 1998–Dec 2012	Deaths, all causes, n (%)	CHD death + fatal stroke, n (%)	CHD death, n (%)	Fatal stroke, n (%)	Earliest of either non-fatal/fatal MI/Stroke, n (%)	Earliest of non-fatal MI or CHD death, n (%)	Earliest of non-fatal/fatal stroke, n (%)	Daily mean temperature °C during events days, mean (SD)^2^	Daily mean temperature °C during control days, mean (SD)^2^	Cold spells of three days during events days, n (%)^2^	Cold spells of three days during control days, n (%)^2^	Cold spells of four days during events days, n (%)^2^	Cold spells of four days during control days, n (%)^2^
January	145 (8.5)	47 (9.0)	31 (7.9)	16 (12.0)	84 (9.1)	48 (7.8)	39 (10.0)	3.6 (3.3)	4.2 (3.0)	14 (16.7)	18 (5.7)	8 (9.5)	14 (4.4)
February	149 (8.7)	51 (9.8)	37 (9.5)	14 (11.0)	93 (10.0)	58 (9.4)	40 (11.0)	4.2 (3.1)	4.3 (3.0)	4 (4.3)	16 (5.6)	3 (3.2)	11 (3.9)
March	154 (9.0)	49 (9.4)	36 (9.2)	13 (10.0)	94 (10.0)	63 (10.0)	39 (10.0)	4.8 (3.3)	6.8 (3.0)	18 (19.1)	8 (2.3)	15 (16.0)	7 (2.0)
April	128 (7.5)	35 (6.7)	25 (6.4)	10 (7.7)	74 (8.0)	45 (7.3)	32 (8.6)	8.0 (2.8)	8.5 (2.7)	1 (1.4)	10 (4.3)	0 (0.0)	4 (1.7)
May	127 (7.4)	33 (6.3)	26 (6.6)	7 (5.4)	77 (8.4)	44 (7.1)	38 (10.0)	10.9 (2.9)	11.7 (2.7)	10 (13.0)	10 (4.1)	4 (5.2)	6 (2.4)
June	128 (7.5)	43 (8.3)	32 (8.2)	11 (8.5)	72 (7.8)	52 (8.4)	24 (6.5)	14.2 (2.4)	14.8 (2.8)	6 (8.3)	10 (4.4)	3 (4.2)	1 (0.4)
July	123 (7.2)	31 (6.0)	23 (5.9)	8 (6.2)	55 (6.0)	35 (5.7)	24 (6.5)	16.0 (2.2)	15.9 (2.4)	0 (0.0)	4 (2.2)	0 (0.0)	0 (0.0)
August	153 (9.0)	43 (8.3)	32 (8.2)	11 (8.5)	73 (7.9)	44 (7.1)	35 (9.4)	16.1 (3.1)	15.9 (2.3)	2 (2.7)	5 (1.8)	0 (0.0)	4 (1.5)
September	124 (7.3)	42 (8.1)	31 (7.9)	11 (8.5)	60 (6.5)	43 (7.0)	20 (5.4)	13.6 (2.4)	14.1 (2.3)	2( 3.3)	2 (0.9)	2 (3.3)	2 (0.9)
October	158 (9.3)	45( 8.6)	36 (9.2)	9 (6.9)	86 (9.3)	62 (10.0)	30 (8.1)	11.3 (2.8)	10.5 (3.0)	2 (2.3)	13 (4.1)	2 (2.3)	7 (2.2)
November	138 (8.1)	40 (7.7)	34 (8.7)	6 (4.6)	59 (6.4)	43 (7.0)	19 (5.1)	7.6 (2.7)	7.0 (3.0)	0 (0.0)	8 (4.2)	0 (0.0)	3 (1.6)
December	178 (10.0)	62 (12.0)	48 (12.0)	14 (11.0)	94 (10.0)	79 (13.0)	32 (8.6)	4.8 (3.2)	4.2 (3.4)	5 (5.3)	10 (3.2)	3 (3.2)	8( 2.6)
Overall year	1705 (100)	521 (100)	391 (100)	130 (100)	921 (100)	616 (100)	372 (100)	9.1 (5.3)	9.3 (5.2)	64 (6.9)	114 (3.6)	40 (4.3)	67 (2.1)
Difference winter vs non-winter (%)^1^	+ 18	+ 36	+ 29	+ 59	+ 34	+ 37	+ 37						

1 By convention, the events per day in the four coldest ‘winter’ months (December, January, February, March for the northern hemisphere), minus the events per day over other, ‘non-winter’ months, all divided by the average number of events per day over the non-winter months, multiplied by 100.

2 The ‘events’ definition used in this column refers to the earliest of fatal/non-fatal stroke or CHD death/non-fatal MI during the study period.

**Table 2b:** PROSPER events, temperature, and cold spells by month.

	Events							TEMPERATURE (°C)		COLD SPELL 3 + DAYS		COLD SPELL 4 + DAYS	
PROSPER follow up period: from Dec 1997–June 2009	Deaths, all causes, n (%)	CHD death + fatal stroke, n (%)	CHD death, n (%)	Fatal stroke, n (%)	Earliest of either non-fatal/fatal MI/Stroke, n(%), Glasgow participants	Earliest of non-fatal MI or CHD death, n (%), Glasgow participants	Earliest of non-fatal/fatal stroke, n (%), Glasgow participants	Daily average temperature °C during events days, mean (SD)^2^	Daily average temperature °C during control days, mean (SD)^2^	Cold spells of three days during events days, n (%)^2^	Cold spells of three days during control days, n(%) ^2^	Cold spells of four days during events days, n(%) a2	Cold spells of four days during control days, n(%) a2
January	253(10.3)	70(8.6)	49(8.4)	21(9.2)	63(8.3)	37(8.5)	28(7.8)	4.7(2.5)	4.4(2.8)	4(6.3)	13(5.8)	2(3.2)	8(3.6)
February	213(8.6)	67(8.3)	52(8.9)	15(6.6)	63(8.3)	39(9.0)	31(8.7)	4.4(2.8)	4.6(2.5)	4(6.3)	6(3.2)	1(1.6)	0(0.0)
March	209(8.5)	86(10.6)	64(11.0)	22(9.6)	65(8.6)	41(9.4)	26(7.3)	5.9(2.2)	6.1(2.8)	1(1.5)	13(5.7)	1(1.5)	11(4.8)
April	208(8.4)	77(9.5)	50(8.6)	27(11.8)	70(9.2)	32(7.4)	38(10.6)	8.4(2.2)	8.5(2.4)	1(1.4)	3(1.3)	0(0.0)	0(0.0)
May	201(8.2)	55(6.8)	41(7.0)	14(6.1)	66(8.7)	35(8.0)	30(8.4)	11.2(2.1)	11.3(2.1)	2(3.0)	4(1.7)	1(1.5)	0(0.0)
June	206(8.4)	68(8.4)	42(7.2)	26(11.4)	69(9.1)	38(8.7)	34(9.5)	13.8(2.1)	13.8(2.2)	3(4.3)	10(4.3)	1(1.4)	5(2.1)
July	204(8.3)	57(7.0)	43(7.4)	14(6.1)	55(7.2)	35(8.0)	26(7.3)	15.2(2.1)	15.3(1.8)	2(3.6)	4(2.1)	2(3.6)	3(1.6)
August	167(6.8)	57(7.0)	40(6.9)	17(7.5)	47(6.2)	28(6.4)	23(6.4)	15.2(1.8)	15.1(1.9)	0(0.0)	3(1.8)	0(0.0)	2(1.2)
September	187(7.6)	58(7.2)	46(7.9)	12(5.3)	59(7.8)	33(7.6)	26(7.3)	13.1(2.5)	13.1(2.1)	0(0.0)	1(0.5)	0(0.0)	1(0.5)
October	206(8.4)	73(9.0)	52(8.9)	21(9.2)	80(10.5)	50(11.5)	35(9.8)	9.7(2.3)	9.7(2.5)	1(1.2)	6(2.2)	1(1.2)	4(1.5)
November	184(7.5)	61(7.5)	45(7.7)	16(7.0)	62(8.2)	27(6.2)	37(10.3)	6.9(3.0)	7.0(2.5)	1(1.6)	2(1.0)	1(1.6)	1(0.5)
December	225(9.1)	81(10.0)	58(10.0)	23(10.1)	60(7.9)	40(9.2)	24(6.7)	4.0(3.0)	4.1(3.1)	0(0.0)	3(1.5)	0(0.0)	1(0.5)
Overall year	2463(100)	810(100)	582(100)	228(100)	760(100)	435(100)	358(100)	9.2(4.6)	9.3(4.6)	19(2.5)	68(2.6)	10(1.3)	36(1.4)
Difference winter vs non-winter (%)^1^	+ 18	+ 23	+ 27	+ 13	+ 1	+ 15	− 11						

1 By convention, the events per day in the four coldest ‘winter’ months (December, January, February, March for the northern hemisphere), minus the events per day over other, ‘non-winter’ months, all divided by the average number of events per day over the non-winter months, multiplied by 100.

2 The ‘events’ definition used in this column refers to the earliest of fatal/non-fatal stroke or CHD death/non-fatal MI during the study period.

**Table 3:** Effect of cold spells on events. BRHS and PROSPER. The statistically significant results are marked in bold.

BRHS follow up period: from Jan 1998–Dec 2012	Cold spell of 3 + days	Cold spell of 3 + days adjusted for mean temperature lag 0–6	Cold spell of 4 + days	Cold spell of 4 + days adjusted for mean temperature lag 0–6
Mortality	RR (95% CI)	RR (95% CI)	RR (95% CI)	RR (95% CI)
All causes of death, n = 1705	1.09 (0.82,1.43)	1.09 (0.81,1.47)	1.14 (0.81,1.62)	1.15 (0.80,1.67)
CHD death + Fatal Stroke, n = 521	**1.62 (1.03,2.55)**	1.62 (0.98,2.67)	**1.92 (1.11,3.33)**	**1.93 (1.06,3.52)**
CHD death, n = 391	**1.76 (1.05,2.95)**	1.70 (0.95,3.02)	**2.41 (1.30,4.48)**	**2.38 (1.21,4.69)**
Fatal Stroke, n = 130	1.24 (0.47,3.24)	1.37 (0.48,3.88)	0.86 (0.24,3.16)	0.91 (0.23,3.64)

*Non-fatal or fatal events*				
Earliest of either non-fatal/fatal MI/Stroke, n = 921	**2.05 (1.49,2.83)**	**1.86 (1.30,2.65)**	**2.21 (1.47,3.32)**	**1.91 (1.23,2.97)**
Earliest of non-fatal MI or CHD death, n = 616	**2.23 (1.51,3.28)**	**2.14 (1.39,3.28)**	**2.86 (1.75,4.69)**	**2.70 (1.58,4.60)**
Earliest of non-fatal/fatal stroke, n = 372	1.69 (0.99,2.89)	1.46 (0.82,2.62)	1.40 (0.69,2.81)	1.13 (0.54,2.38)



PROSPER follow up period: from Dec 1997–June 2009	Cold spell of 3 + days	Cold spell of 3 + days adjusted for mean temperature lag 0–6	Cold spell of 4 + days	Cold spell of 4 + days adjusted for mean temperature lag 0–6
Mortality	RR (95% CI)	RR (95% CI)	RR (95% CI)	RR (95% CI)
All causes of death, n = 2463	1.28 (0.99,1.65)	1.27 (0.96,1.67)	1.16 (0.81,1.64)	1.12 (0.77,1.62)
CHD death + Fatal Stroke, n = 810	1.13 (0.74,1.73)	1.11 (0.70,1.77)	0.72 (0.37,1.39)	0.67 (0.34,1.33)
CHD death, n = 582	1.10 (0.67,1.82)	1.14 (0.66,1.96)	0.74 (0.34,1.58)	0.73 (0.33,1.62)
Fatal Stroke, n = 228	1.22 (0.53,2.78)	1.05 (0.43,2.59)	0.68 (0.19,2.40)	0.53 (0.14,2.01)

*Non-fatal or fatal events*				
Earliest of either non-fatal/fatal MI/Stroke, Glasgow participants, n = 760	0.99 (0.59,1.65)	0.91 (0.53,1.57)	1.04 (0.52,2.10)	0.95 (0.46,1.98)
Earliest of non-fatal MI or CHD death, Glasgow participants, n = 435	1.23 (0.67,2.27)	1.07 (0.55,2.08)	1.25 (0.56,2.78)	1.06 (0.46,2.48)
Earliest of non-fatal/fatal stroke, Glasgow participants, n = 358	0.75 (0.33,1.72)	0.75 (0.32,1.79)	1.16 (0.43,3.18)	1.22 (0.43,3.50)

**Table 4:** Effect of temperature on events for BRHS and PROSPER. The statistically significant results are marked in bold.

BRHS follow up period: from Jan 1998–Dec 2012	Decrease of 1 °C in mean temperature, cumulative lag 0–3	Decrease of 1 °C in mean temperature, cumulative lag 0–3 adjusted for cold spell of 3 + days	Decrease of 1 °C in mean temperature, cumulative lag 0–6	Decrease of 1 °C in mean temperature, cumulative lag 0–6 adjusted for cold spell of 3 + days
Mortality	RR (95% CI)	RR (95% CI)	RR (95% CI)	RR (95% CI)
All causes of death, n = 1705	0.99 (0.97,1.02)	0.99 (0.96,1.02)	1.00 (0.98,1.03)	1.00 (0.97,1.03)
CHD death + Fatal Stroke, n = 521	1.02 (0.98,1.06)	1.00 (0.96,1.05)	1.02 (0.97,1.07)	1.00 (0.95,1.05)
CHD death, n = 391	1.03 (0.98,1.08)	1.01 (0.96,1.06)	1.03 (0.98,1.08)	1.00 (0.94,1.06)
Fatal Stroke, n = 130	0.99 (0.91,1.07)	0.98 (0.89,1.07)	0.99 (0.88,1.08)	0.99 (0.88,1.09)

*Non-fatal or fatal events*				
Earliest of either non-fatal/fatal MI/Stroke, n = 921	**1.06(1.03,1.08)**	**1.03(1.00,1.07)**	**1.05(1.01,1.08)**	1.02(0.98,1.06)
Earliest of non-fatal MI or CHD death, n = 616	**1.04(1.01,1.08)**	1.01(0.97,1.05)	**1.04(1.00,1.08)**	1.01(0.96,1.05)
Earliest of non-fatal/fatal stroke, n = 372	**1.07(1.02,1.12)**	**1.06(1.01,1.11)**	1.05(0.99,1.10)	1.03(0.97,1.09)



PROSPER follow up period: from Dec 1997–June 2009	Decrease of 1 °C in mean temperature, cumulative lag 0–3	Decrease of 1 °C in mean temperature, cumulative lag 0–3 adjusted for cold spell of 3 + days	Decrease of 1 °C in mean temperature, cumulative lag 0–6	Decrease of 1 °C in mean temperature, cumulative lag 0–6 adjusted for cold spell of 3 + days
Mortality	RR (95% CI)	RR (95% CI)	RR (95% CI)	RR (95% CI)
All causes of death, n = 2463	1.00 (0.98,1.03)	0.99 (0.97,1.02)	1.01 (0.98,1.04)	1.00 (0.97,1.03)
CHD death + Fatal Stroke, n = 810	1.01 (0.97,1.05)	1.00 (0.96,1.05)	1.01 (0.96,1.06)	1.00 (0.96,1.05)
CHD death, n = 582	1.00 (0.96,1.05)	1.00 (0.95,1.05)	1.00 (0.94,1.05)	0.99 (0.93,1.05)
Fatal Stroke, n = 228	1.02 (0.95,1.10)	1.01 (0.93,1.10)	1.04 (0.96,1.13)	1.04 (0.95,1.14)

*Non-fatal or fatal events*				
Earliest of either non-fatal/fatal MI/Stroke, Glasgow participants, n = 760	1.01 (0.97,1.05)	1.01 (0.97,1.06)	1.02 (0.97,1.07)	1.02 (0.97,1.07)
Earliest of non-fatal MI or CHD death, Glasgow participants, n = 435	1.03 (0.98,1.09)	1.03 (0.97,1.10)	1.04 (0.98,1.11)	1.04 (0.97,1.11)
Earliest of non-fatal/fatal stroke, Glasgow participants, n = 358	0.99 (0.93,1.05)	1.00 (0.94,1.06)	0.99 (0.93,1.06)	1.00 (0.93,1.07)

**Table 5:** BRHS participants. Interactions between cold spells of different durations and individual characteristics on earliest of either non-fatal/fatal MI/Stroke. P-values < 0.05 are marked in bold.

Earliest of either non-fatal/fatal MI/Stroke	N	Cold spell 3 + days RR (95% CI)	p-Value for interaction	Cold spell 4 + days RR (95% CI)	p-Value for interaction
Age < 70	428	1.83 (1.12,2.99)	0.547	1.93 (1.02,3.64)	0.579
Age > = 70	493	2.24 (1.46,3.43)		2.44 (1.43,4.15)	
Non-Manual social class	397	2.69 (1.64,4.40)	0.229	2.73 (1.46,5.10)	0.520
Manual social class	488	1.80 (1.16,2.78)		2.07 (1.20,3.59)	
Previous stroke/MI, No	763	2.07 (1.47,2.91)	0.867	2.25 (1.45,3.47)	0.846
Previous stroke/MI, Yes	158	1.90 (0.72,5.03)		1.99 (0.63,6.26)	
Normal weight (BMI 18.5–25)	261	1.81 (0.99,3.30)	0.625	2.31 (1.08,4.93)	0.898
Underweight or overweight	653	2.16 (1.48,3.16)		2.17 (1.34,3.52)	
Diabetes, No	784	2.05 (1.45,2.90)	0.977	2.15 (1.36,3.38)	0.767
Diabetes, Yes	137	2.07 (0.90,4.78)		2.51 (1.00,6.30)	
COPD: NO (FEV1/FEVC ≥ 70%)	693	1.99 (1.38,2.87)	0.884	2.18 (1.38,3.44)	0.893
COPD: YES (FEV1/FEVC < 70%)	214	2.11 (1.06,4.17)		2.03 (0.80,5.17)	
Physical activity: light/occasional/inactive	525	2.04 (1.29,3.22)	0.756	1.80 (1.03,3.15)	0.428
Physical activity: from moderate to vigorous	352	1.83 (1.13,2.99)		2.55 (1.33,4.89)	
Non-smoker	233	0.97 (0.45,2.10)	**0.034**	0.58 (0.17,1.99)	**0.019**
Smoker or ex-smoker	684	2.44 (1.70,3.50)		2.79 (1.77,4.38)	
Occasional or non-drinker	390	1.41 (0.82,2.42)	0.080	1.34 (0.70,2.58)	**0.039**
Light/Moderate/Heavy drinker	506	2.59 (1.71,3.92)		3.29 (1.91,5.67)	
Owner occupier	745	2.16 (1.51,3.09)	0.317	2.39 (1.50,3.81)	0.352
Renting from the local authority	112	1.30 (0.52,3.27)		1.37 (0.47,4.01)	
Central heating: Yes	807	2.18 (1.55,3.07)	0.329	2.48 (1.60,3.83)	0.182
Central Heating: No	114	1.32 (0.51,3.42)		1.04 (0.31,3.45)	
Double glazing: No	404	2.20 (1.44,3.35)	0.626	2.77 (1.63,4.71)	0.199
Double glazing: Yes/in part	517	1.87 (1.13,3.07)		1.60 (0.84,3.07)	
Married	721	2.17 (1.49,3.16)	0.378	2.50 (1.55,4.01)	0.195
Single/divorced/separated/widowed/other	160	1.53 (0.77,3.03)		1.24 (0.49,3.18)	
Retired	106	1.94 (1.37,2.76)	0.716	2.04 (1.31,3.17)	0.873
Employed	778	1.60 (0.61,4.23)		1.81 (0.45,7.27)	
Living alone	128	1.88 (0.89,3.98)	0.839	1.84 (0.68,4.98)	0.719
Living with others	765	2.05 (1.42,2.94)		2.25 (1.42,3.54)	
Car owner: Yes	705	2.44 (1.70,3.51)	**0.035**	2.84 (1.77,4.55)	0.053
Car owner: No	194	0.94 (0.42,2.11)		1.01 (0.40,2.58)	
Regular use of aspirin: No	557	1.78 (1.17,2.71)	0.299	1.76 (1.03,3.01)	0.182
Regular use of aspirin: Yes	358	2.52 (1.53,4.14)		3.11 (1.64,5.88)	
Use of Beta Blockers: No	771	2.00 (1.42,2.83)	0.721	1.99 (1.27,3.12)	0.256
Use of Beta Blockers: Yes	150	2.37 (1.00,5.60)		3.77 (1.38,10.29)	
Use of statin: No	820	2.11 (1.51,2.96)	0.573	2.32 (1.51,3.55)	0.506
Use of statin: Yes	101	1.53 (0.52,4.48)		1.42 (0.36,5.66)	
Region: South/Midlands	413	2.11 (1.28,3.45)	0.892	1.53 (0.78,3.00)	0.164
Region: North/Scotland	508	2.01 (1.32,3.07)		2.79 (1.66,4.69)	
Non winter months	556	1.25 (0.77,2.03)	**0.004**	1.42 (0.69,2.91)	0.128
Winter months (Dec–Mar)	365	3.28 (2.09,5.13)		2.81 (1.70,4.64)	

## Appendix A Supplementary data

Supplementary material Image 1
