# Supplementary material for: Effect of cold spells and their modifiers on cardiovascular disease events: Evidence from two prospective studies
Source: Int J Cardiol. 2016 Sep 1;218:275–83. doi: 10.1016/j.ijcard.2016.05.012 (PMC4917887; doi:10.1016/j.ijcard.2016.05.012)
Supplement: Supplementary file 1 — Supplementary material [file mmc1.docx]

Appendix A. Supplementary data

*BRHS and PROSPER methods* *and participants*

*BRHS*

The design of the British Regional Heart Study (BRHS) has been described in detail [1]. To summarise, the BRHS is a prospective cohort of 7735 men recruited from a single local primary care centre in each of 24 British towns in 1978-80 (age 40-59 years). Between January 1998 and March 2000, after an average of 20 years, the survivors aged 60–79 years attended for follow-up clinical measurements (anthropometry, physiological, blood and urine sample) and completed a detailed questionnaire [2]. For the present analysis participants were followed-up for incidence of CVD from 1998 until the end of 2012. Only BRHS men who did not change town of residence between 1998 and 2012 were included, in order to avoid misclassification of exposure to ambient temperature. The physical measurements used in the present work were carried out by a team of specially trained nurses who rotated through different workstations to ensure that each nurse assessed an equal number of participants within each town. Measurements collected were BMI (kg/m2), forced expiratory volume in 1 second, (FEV_1_), and Force Vital capacity, FVC. BMI was classified in 4 categories (<18.5, 18.5-24.9, 25-29.9, ≥30); the FEV1/FVC ratio was used in the diagnosis of chronic obstructive pulmonary disease ([FEV_1_/FVC%] <70 vs ≥70). [2]

Age was calculated at the date that the questionnaire was completed, while occupational social class was classified as manual or non-manual [3]. For the present analysis we also included questions on (i) physical health (prevalence of previous non-fatal stroke or MI, and diabetes); (ii) personal risk factors (smoking status, alcohol consumption, self-reported physical activity score ); (iii) house characteristics (central heating availability, double glazing, and ownership of the house); (iv) personal circumstances (marital status, working status, living alone vs not, and car ownership); and (v) use of medication (aspirin, warfarin, beta-adrenoceptor blocking drugs, and statin).

*PROSPER*

The PROSPER study design has been previously described [4, 5]. The study is a prospective cohort which includes three European coordinating centres (Glasgow, Scotland; Cork, Ireland; and Leiden, The Netherlands). PROSPER was originally constructed as a double blind randomized trial of pravastatin versus placebo. Approximately 50% of the study population have evidence of vascular disease, and the other 50% are at high risk for vascular disease because they have one major vascular risk factors (hypertension, cigarette smoking, or diabetes mellitus [5]. The study recruited subjects with a history of vascular disease, or, if that was not present, who satisfied at least one of the following conditions: to be smokers, with hypertension, and diabetes. The subjects living in the three towns and the surrounding areas were screened and enrolled between December 1997 and May 1999 (n=5804, 2806 men and 2998 women) when aged 70–82 [6]. Differently from BRHS participants, during the follow-up period a change in town of residence was not available. The follow up was initially over an average of 3.2 years and it has subsequently been extended for mortality for the whole study and hospital admissions for the Scottish participants. The physical measurement used in this work was BMI (kg/m2), classified in 4 categories (<18.5, 18.5-24.9, 25-29.9, ≥30). For the present analysis we also included questions on (i) physical health (prevalence of previous non-fatal stroke or MI, and diabetes); (ii) personal risk factors (smoking status, alcohol consumption); (iii) personal circumstances (living alone vs not); and (v) use of medication at baseline (aspirin, warfarin, and statin).

*Definition of fatal and non-fatal CVD events*

*Non-fatal events*

To summarise, non-fatal stroke events were those that produced a neurological deficit that was present for >24 hours; non-fatal MI was defined by the presence of at least two of severe prolonged chest pain, electrocardiograph (ECG) evidence of MI, and cardiac enzymes changes consistent with MI. Both non-fatal stroke and non-fatal MI events were ascertained by regular reviews of general practitioner records in the BRHS; for the PROSPER Scottish population non-fatal events were determined by established record-linkage methods through hospital admissions [7, 8].

*Fatal events*

For both BRHS and PROSPER fatal cases were ascertained through National Health Services Central Registers (in Scotland through General Register Office (GRO) via Information Services Division). Death certificates were extracted from the databases using appropriate International Classification of Diseases-Ninth Revision codes 430–438 for stroke and 410–414 for MI. Some BRHS fatal events may have been preceded by a non-fatal event in the 28 days prior to death [9], but date of death was used for this analysis.

*Climatic data*

The UK Meteorological Office (www.metoffice.gov.uk) provided daily maximum and minimum temperatures for BRHS and PROSPER UK towns (34 UK towns and Glasgow). The Royal Netherlands Meteorological Institute (www.knmi.nl) and the Irish Meteorological Service (www.met.ie) provided temperature data for Leiden and Cork respectively. The mean temperature of the day was calculated as average of maximum and minimum temperatures. Specifically, the participants resident in 24 BRHS towns were matched with the closest of the 34 weather stations available via post code of residence (average distance of about 10 kilometres). PROSPER participants living in the three towns and the surrounding areas were matched with the Glasgow, Cork and Leiden meteorological station.

*Definition of cold spell*

As previous studies reported, the relationship between the occurrence of cold episodes and events is based on (i) duration (number of days of cold spell); (ii) intensity or frequency (influenced by the chosen threshold for temperature, such as the 10^th^ percentile for that month); (iii) lag period (time lapse between the cold episode and the registration of an outcome) and (iv) acclimatisation (the fact that individuals adapt to their local weather conditions) [10]. In these analyses daily mean temperature was dichotomized according to the 10th percentile of the historical monthly distribution of each weather station over the period 1998-2012. We considered days below the 10^th^ percentile as cold days. Using a weather station-specific and monthly percentile, rather than a common cut-off at a given temperature, assumed that individuals adapt to their local weather conditions [11].

In this study the definition of cold spell meets the following criteria: for each one of the 34 BRHS meteorological stations (plus Glasgow, Cork and Leiden for PROSPER), cold spell episodes were defined as at least 3 and 4 consecutive days prior to the day of interest (lag 0-3 and lag 0-4) with mean temperatures below the 10^th^ percentile of the monthly distribution of the reference period.

eFigure1 - Percentage of events occurred by month in the BRHS and the PROSPER during the study period (1997-2012). The total number of events during the study period is reported in the legend box for both studies


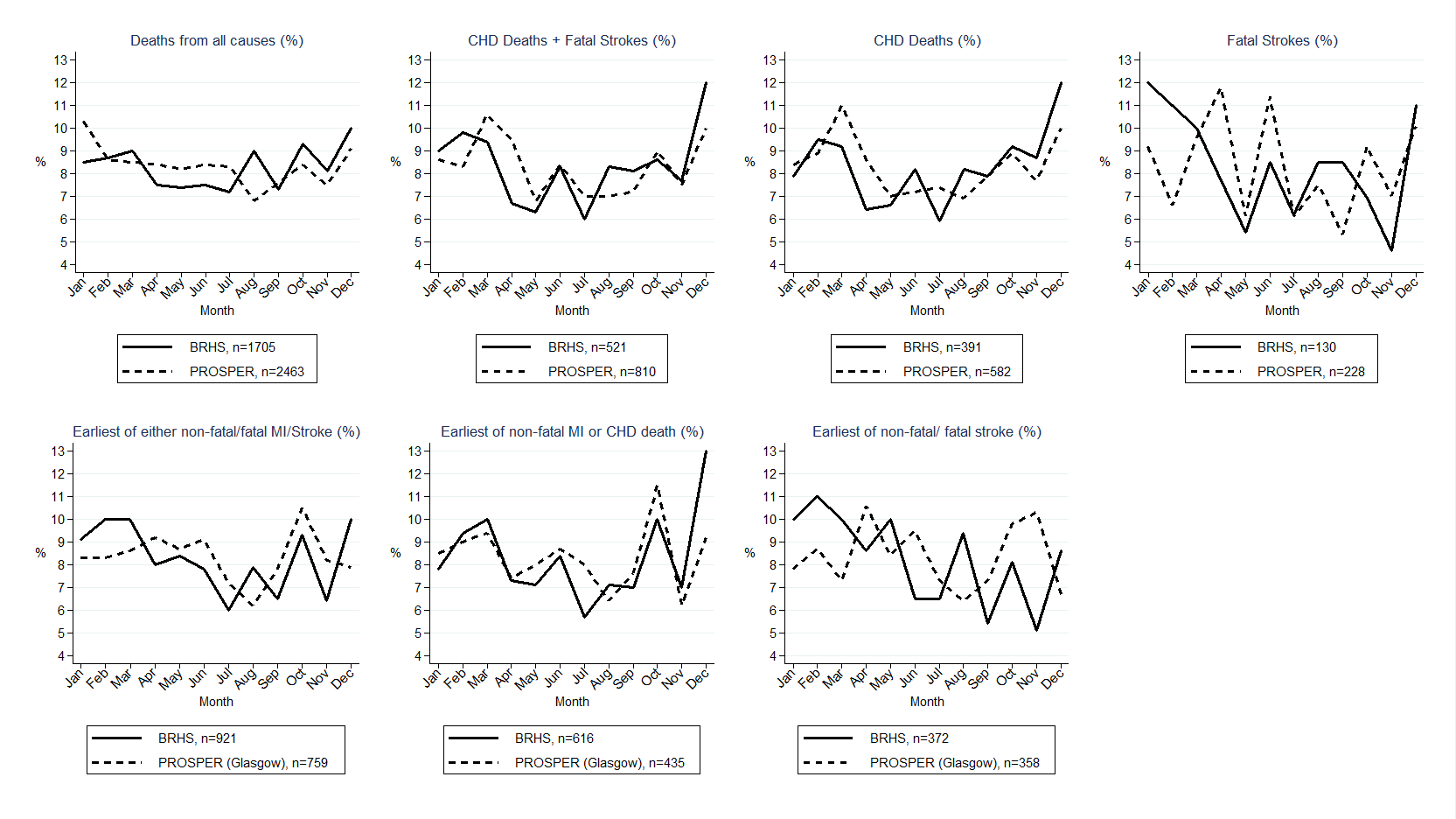


References

[1] Shaper AG, Pocock SJ, Walker M, Cohen NM, Wale CJ, Thomson AG. British Regional Heart Study: cardiovascular risk factors in middle-aged men in 24 towns. BMJ. 283 (1981) 179-86.

[2] Walker M, Whincup P, Shaper A. The British Regional Heart Study 1975–2004. International Journal of Epidemiology. 33 (2004) 1185-92.

[3] Ramsay SE, Whincup PH, Papacosta O, Morris RW, Lennon LT, Wannamethee SG. Inequalities in heart failure in older men: prospective associations between socioeconomic measures and heart failure incidence in a 10-year follow-up study. European heart journal. 35 (2014) 442-7.

[4] Shepherd J, Blauw GJ, Murphy MB, Bollen ELEM, Buckley BM, Cobbe SM, et al. Pravastatin in elderly individuals at risk of vascular disease (PROSPER): a randomised controlled trial. The Lancet. 360 (2002) 1623-30.

[5] Shepherd J, Blauw GJ, Murphy MB, Cobbe SM, Bollen ELEM, Buckley BM, et al. The design of a prospective study of pravastatin in the elderly at risk (PROSPER). The American Journal of Cardiology. 84 (1999) 1192-7.

[6] Loeffen R, Winckers K, Ford I, Jukema JW, Robertson M, Stott DJ, et al. Associations Between Thrombin Generation and the Risk of Cardiovascular Disease in Elderly Patients: Results From the PROSPER Study. The Journals of Gerontology Series A: Biological Sciences and Medical Sciences. 70 (2014) 982-8.

[7] Kendrick S, Clarke J. The Scottish Record Linkage System. Health bulletin. 51 (1993) 72-9.

[8] Lloyd SM, Stott DJ, de Craen AJ, Kearney PM, Sattar N, Perry I, et al. Long-term effects of statin treatment in elderly people: extended follow-up of the PROspective Study of Pravastatin in the Elderly at Risk (PROSPER). PLoS One. 8 (2013) e72642.

[9] Wannamethee G, Whincup PH, Shaper AG, Walker M, MacFarlane PW. Factors determining case fatality in myocardial infarction "who dies in a heart attack"? Br Heart J. 74 (1995) 324-31.

[10] Monteiro A, Carvalho V, Gois J, Sousa C. Use of "Cold Spell" indices to quantify excess chronic obstructive pulmonary disease (COPD) morbidity during winter (November to March 2000-2007): case study in Porto. International journal of biometeorology. 57 (2013) 857-70.

[11] Medina-Ramon M, Schwartz J. Temperature, temperature extremes, and mortality: a study of acclimatisation and effect modification in 50 US cities. Occupational and environmental medicine. 64 (2007) 827-33.
